# Drosophila as a Model to Study Cellular Communication Between the Hematopoietic Niche and Blood Progenitors Under Homeostatic Conditions and in Response to an Immune Stress

**DOI:** 10.3389/fimmu.2021.719349

**Published:** 2021-08-16

**Authors:** Ismaël Morin-Poulard, Yushun Tian, Nathalie Vanzo, Michèle Crozatier

**Affiliations:** MCD/UMR5077, Centre de Biologie Intégrative (CBI), Toulouse, France

**Keywords:** *Drosophila*, lymph gland, niche, hematopoiesis, immune stress

## Abstract

In adult mammals, blood cells are formed from hematopoietic stem progenitor cells, which are controlled by a complex cellular microenvironment called “niche”. *Drosophila melanogaster* is a powerful model organism to decipher the mechanisms controlling hematopoiesis, due both to its limited number of blood cell lineages and to the conservation of genes and signaling pathways throughout bilaterian evolution. Insect blood cells or hemocytes are similar to the mammalian myeloid lineage that ensures innate immunity functions. Like in vertebrates, two waves of hematopoiesis occur in *Drosophila*. The first wave takes place during embryogenesis. The second wave occurs at larval stages, where two distinct hematopoietic sites are identified: subcuticular hematopoietic pockets and a specialized hematopoietic organ called the lymph gland. In both sites, hematopoiesis is regulated by distinct niches. In hematopoietic pockets, sensory neurons of the peripheral nervous system provide a microenvironment that promotes embryonic hemocyte expansion and differentiation. In the lymph gland blood cells are produced from hematopoietic progenitors. A small cluster of cells called Posterior Signaling Centre (PSC) and the vascular system, along which the lymph gland develops, act collectively as a niche, under homeostatic conditions, to control the balance between maintenance and differentiation of lymph gland progenitors. In response to an immune stress such as wasp parasitism, lymph gland hematopoiesis is drastically modified and shifts towards emergency hematopoiesis, leading to increased progenitor proliferation and their differentiation into lamellocyte, a specific blood cell type which will neutralize the parasite. The PSC is essential to control this emergency response. In this review, we summarize *Drosophila* cellular and molecular mechanisms involved in the communication between the niche and hematopoietic progenitors, both under homeostatic and stress conditions. Finally, we discuss similarities between mechanisms by which niches regulate hematopoietic stem/progenitor cells in *Drosophila* and mammals.

## Introduction

Hematopoiesis is the process that leads to the constant formation of blood cells throughout metazoan life. In vertebrates, hematopoietic stem and progenitor cells (HSPCs) give rise to all blood cell types. In adults, HSPCs are found in the bone marrow, and its microenvironment, termed ‘niche’, ensures hematopoietic homeostasis by controlling the proliferation and differentiation of HSPCs, both under normal conditions and in response to a stress such as infection or systemic inflammation (1–4). The ‘niche’ concept was proposed in 1978 by R. Schofield (5) and refers to the cellular context that maintains and regulates HSPC self-renewal and differentiation. The bone marrow hematopoietic niche is now described as a complex multicellular network that supports HSPCs, either *via* direct adhesive interactions or *via* the secretion of many different factors acting in a paracrine manner to control their localization, maintenance, proliferation and differentiation. At least two anatomically distinct HSPC niches exist in the bone marrow. Imaging studies indicate that HSPCs localize around arterioles in the endosteal area, which is in close proximity to the bone surface and is called the endosteal niche (6), and around sinusoids located in the inner bone marrow and called the vascular niche (7–12). Recent advances in single cell technologies allowed the identification of various populations of niche cells with distinct transcriptional profiles, revealing the huge complexity of the cell population within the bone marrow hematopoietic niches (8, 13–16). Furthermore, the HSPC pool itself is heterogeneous, raising the possibility that distinct and specific niche cell types control subsets of HSPCs (6, 17, 18).

For 15 years, *Drosophila melanogaster* has proven to be a suitable model organism to investigate the mechanisms controlling hematopoiesis, based both on limited blood cell lineages and on functional parallels with the vertebrate system. In flies, blood/immune cells are called hemocytes and are related to vertebrate myeloid cells

**Figure 1:**
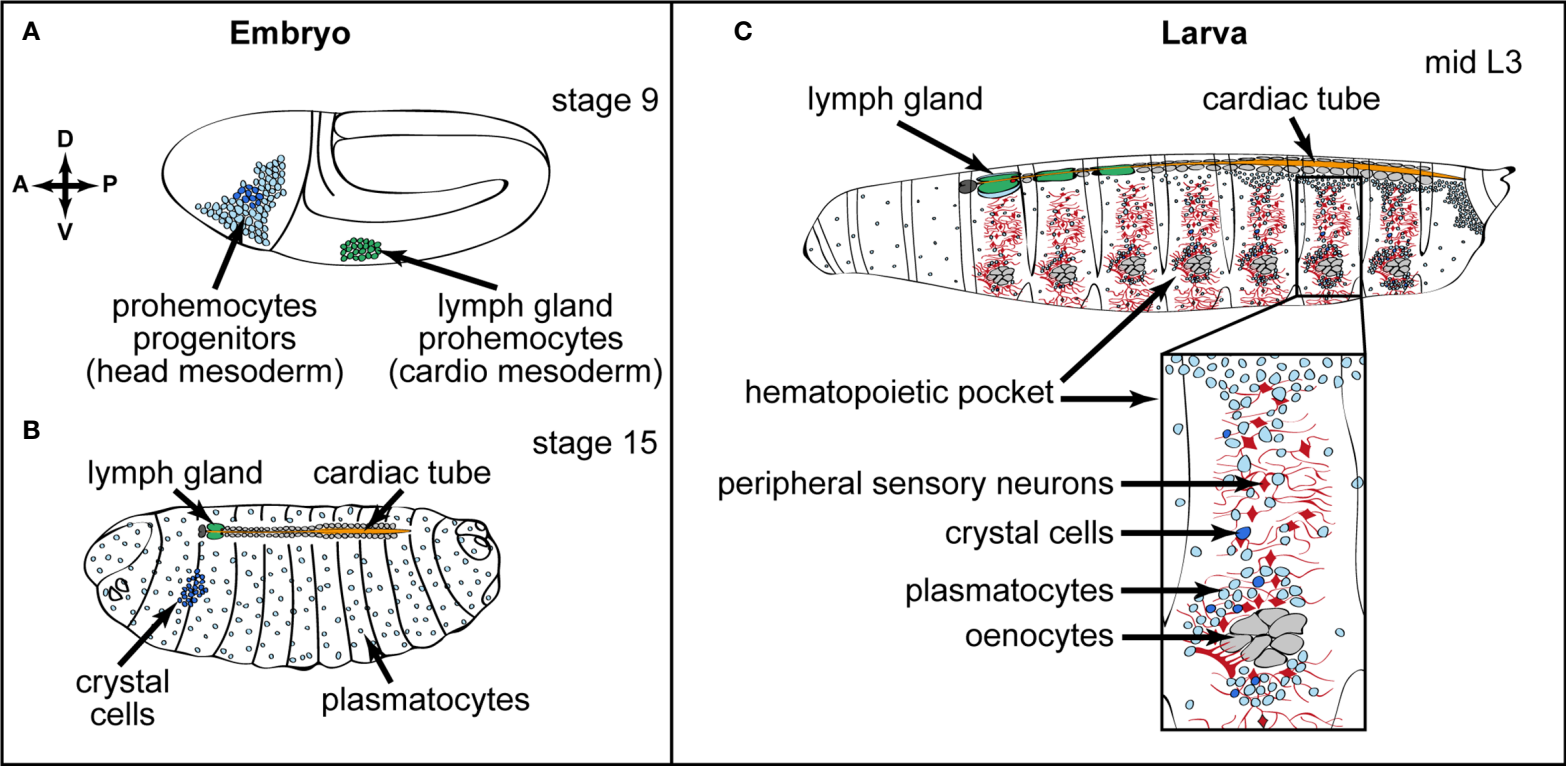
Embryonic and larval hematopoiesis. **(A, B)** Embryonic hemocytes (blood cells) originate from the head mesoderm in the embryo and differentiate into plasmatocytes (macrophages, light blue) and a small number of crystal cells (dark blue). Lymph gland progenitors (green) are specified from the thoracic cardiogenic mesoderm in the embryo **(A)** Anterior (A)/Posterior (P) and Dorsal (D)/Ventral (V) axes are indicated. **(B)** At the end of embryogenesis, crystal cells remain clustered in the anterior part, whereas plasmatocytes are dispersed throughout the embryo. The lymph gland is composed of one pair of lobes and is localized at the anterior part of the dorsal vessel/cardiac tube. **(C)** In third instar larvae, plasmatocytes (light blue) and crystal cells (dark blue) of embryonic origin are found in circulation and colonizing local microenvironments, in particular the hematopoietic pockets, where they expand. Close up of a hematopoietic pocket where neurons are in red, oenocytes in grey, and plasmatocytes and crystal cells in light and dark blue, respectively. Activin-β produced by PNS neurons promotes plasmatocyte proliferation and adhesion. The lymph gland (green) is composed of several pairs of lobes aligned along the cardiac tube.

*Drosophila* hematopoiesis occurs in two waves during development (19, 20). The first wave takes place during embryogenesis (21). A cluster of cells derived from the head mesoderm gives rise to hematopoietic progenitors, which differentiate into plasmatocytes and crystal cells. Plasmatocytes, which are involved in phagocytosis of cellular debris and pathogens, are equivalent to mammalian macrophages (21–24). Crystal cells contain crystalline inclusions of prophenoloxidases, which are required for the synthesis of melanin (25, 26) and are involved in clotting and wound healing (27–29). These hemocytes of embryonic origin persist in larval and adult stages (**Figures 1A, B** and (30–34). The second wave of hematopoiesis takes place in larval stages at two distinct hematopoietic sites: the hematopoietic pockets and the hematopoietic organ called the lymph gland. Hematopoietic pockets are aggregate of embryo-derived hemocytes segmentally repeated in epidermal-muscular clusters underneath the larval cuticle (**Figure 1C**). In addition, *de novo* blood cell specification occurs in the lymph gland (see below) from hematopoietic progenitors and give rise to plasmatocytes, crystal cells and a third blood cell type called lamellocyte. Lamellocytes are not found in larvae under normal conditions, but they massively differentiate in response to an immune stress such as wasp parasitism. Lamellocytes are required for the encapsulation of foreign bodies too large to be engulfed by phagocytosis (29, 35). The lymph gland is localized dorsally, in close association with the *Drosophila* dorsal vessel, which is the vascular system. At metamorphosis, the lymph gland disrupts and all cells are released into the circulation (29, 36). Both embryo and lymph gland-derived blood cells are present in the adult fly and accumulate in the respiratory epithelia and fat body (33, 34, 37). Their numbers continuously decrease with aging (29, 38), and whether adult flies are able to produce new blood cells is currently under debate. Ghosh et al. identified active hematopoietic hubs, localized in the abdomen, and supporting hematopoiesis in adults (34). However, this conclusion is strongly questioned by a recent analysis which studies hemocytes localized in the head and thorax regions and where no indication of *de novo* blood cell production was observed, even after bacterial infections (37). Since recent single cell RNAseq analyses identify different hemocyte populations (39–42), it is possible that hemocytes characterized in these two distinct locations might have a different potential. This point deserves further investigation.

While no data indicate that embryonic hematopoiesis is niche-dependent, several studies established that larval hematopoiesis is under the control of distinct niches. In this review, we will give an overview of the various *Drosophila* hematopoietic niches identified so far and of the molecular cascades that regulate the communication between niche cells and progenitors, both under homeostatic and immune stress conditions. 

## Neurons as a Microenvironment Controlling Embryonic-Derived Hemocytes in Hematopoietic Pockets

At larval stages, most embryonic-derived hemocytes are differentiated macrophages. They are either circulating in the hemolymph or residing in clusters, which are segmentally repeated along the larval body wall and called hematopoietic pockets (29, 43, 44) (**Figure 1C**). There is a continuous and dynamic exchange between circulating and resident/pocket macrophages (44–47). Large hepatocyte-like cells called oenocytes, and sensory neurons from the peripheral neuronal system (PNS), are in close contact with resident macrophages in the hematopoietic pockets (**Figure 1C**). A subset of sensory neurons that produces Activin-β, a ligand of the TGF-β family, regulates their proliferation and adhesion to hematopoietic pockets (48). It should be emphasized that the neuronal niche in hematopoietic pockets and the niche in the lymph gland (see below) have distinct functions. While the neuronal niche is regulating differentiated macrophages, the niche in the lymph gland is controlling both differentiated hemocytes and hematopoietic progenitors. In vertebrate, tissue-resident macrophages regulate tissue homeostasis and contribute to inflammation (49, 50). Resident macrophage proliferation is strongly dependent on the tissue microenvironment, and whether vertebrate neuronal sensing, as described in *Drosophila*, regulates locally macrophage behavior remains to be addressed.

Finally, several studies report on the plasticity of embryonic-derived hemocytes. Within hematopoietic pockets, plasmatocytes can trans differentiate into crystal cells (51, 52). Furthermore, embryonic-derived hemocytes can also give rise to lamellocytes following parasitism (32, 53–56). A puzzling question was whether signals from the neuronal niche might also regulate blood cell plasticity in hematopoietic pockets. A recent study established that in hematopoietic pockets localized at the caudal end of the larva, the trans differentiation of macrophages into crystal cells is promoted by the neuronal activity of a specific subset of oxygen sensing neurons (52). This study establishes that environmental conditions, such as oxygen levels, control *in vivo* blood cell trans differentiation. Whether neuronal control of blood cell trans differentiation in response to environmental conditions is conserved during evolution, deserves further investigation.

## The PSC Acts as a Niche to Control Lymph Gland Hematopoiesis

**Figure 2:**
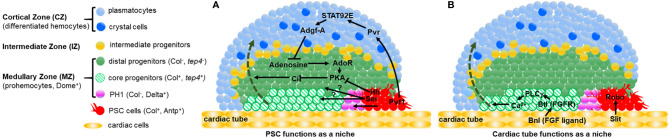
Two niches control lymph gland homeostasis. **(A, B)** Schematic representation of third instar larva lymph gland anterior lobes. The medullary zone (MZ) contains three types of progenitors: distal progenitors and core progenitors are in green and hatched green, respectively, and the PH1 is in pink. Intermediate progenitors are in yellow, plasmatocytes and crystal cells in the cortical zone (CZ) are in light and dark blue, respectively. The PSC and the cardiac tube/vascular system are in red and orange, respectively. **(A)** Differentiated hemocytes result from progenitors’ differentiation (green dashed arrow) In a wildtype (WT) lymph gland, under homeostatic conditions, the PSC regulates the maintenance of a subset of MZ progenitors. Hedgehog (Hh) is required for maintaining distal progenitors. PSC signals required for controlling PH1 remain to be identified, as well as the progenitor subset controlled by Ser expressed in the PSC. Pvf1 secreted by the PSC, controls progenitor maintenance *via* differentiated hemocytes. **(B)** The cardiac tube corresponds to a second niche present in the lymph gland. The FGF ligand Branchless (Bnl) activates its receptor Breathless (Btl) in progenitors. Btl-FGF activation regulates intracellular Ca^2+^ levels *via* PLCγ, and controls the maintenance of core progenitors and in turn the whole progenitor pool. The ligand Slit produced by cardiac cells activates its Robo receptors in the PSC. Robo signaling controls PSC cell clustering and proliferation.

In third instar larvae, the mature lymph gland is composed of paired lobes: one primary pair and several secondary pairs. The anterior lobes, which are the largest in size contain progenitors, differentiating hemocytes and mature blood cells, while posterior lobes are composed of a heterogeneous population of progenitors, which do not undergo terminal differentiation (23, 36, 57, 58). Each anterior lobe is divided into several zones (**Figure 2A**). A central zone, called the medullary zone (MZ), contains tightly packed blood cell progenitors (prohemocytes) characterized by the expression of the Janus kinase/signal transducer and activator of transcription (JAK/STAT) receptor *domeless* (*dome*) (23, 59). Recently, the most internally localized subpopulation of MZ progenitors was further characterized by expression of specific markers such as the Thioester-containing protein-4 (Tep4) and Col (60). This subpopulation is defined as “core progenitors”. The neighboring progenitors lacking *tep4* and *col* expression are called “distal progenitors” (61). Recent advances in single cell technologies established the transcriptional profiles of lymph gland cells under homeostatic conditions and at various developmental time points (41). The molecular signatures, provided by single cell transcriptomic analysis, define an additional prohemocyte sub-cluster called PH1 (prohemocyte 1). At the periphery of anterior lobes, the cortical zone (CZ) is composed of differentiated blood cells that can be identified through the expression of specific markers for plasmatocytes and crystal cells. Between the MZ and the CZ, cells undergo the transition from progenitors to specified blood cells and correspond to intermediate progenitors. They simultaneously express markers for prohemocytes and for early differentiating cells [**Figure 2A** and (59)]. At the posterior end of the primary lobe is the PSC, identified by its expression of the Notch ligand Serrate (Ser) (23, 62), the homeobox protein Antennapedia (Antp) (63) and high levels of Collier/Knot (Col/Kn), an orthologue of mammalian Early B cell factor (EBF) (57, 64). In late third instar larvae the mature PSC is composed of 30-40 cells [**Figure 2A** and (57, 63, 64)] and it plays a role similar to a niche to control lymph gland homeostasis.

Most studies on the PSC’s role as a niche were performed in third instar larvae. The number of PSC cells is tightly controlled and several intrinsic and extrinsic factors regulate their proliferation. Since several recent reviews report on genes and molecular mechanisms that control PSC cell numbers, we shall not develop this specific issue but provide a table summarizing the information [see **Table 1** and reviews by (19, 20, 77)]. PSC cells produce cytoplasmic processes called filopodia that extend over 2 to 3 cell diameters. An interesting possibility is that filopodia could be engaged in direct cellular contacts between PSC cells and MZ progenitors (63, 64).

**Table 1:** Genes and pathways involved in controlling the number of PSC cells and their cohesion.

Gene	Cell type	Genetic conditions	Function	References
Collier/knot	PSC	LOF (*col* RNAi)	Reduces PSC cell number	(65)
Wnt/Wingless	PSC	LOF (UAS-Dfz2^DN^) GOF (UAS-wg)	Promotes PSC cell proliferation	(66)
BMP / Decapentaplegic	PSC	LOF (*dpp* RNAi; UAS-tkv ^DN^ )	Inhibits PSC cell proliferation	(65)
Dally-like	Not determined	*dlp* mutant	Reduces PSC cell number	(65)
Dmyc	PSC	LOF (d*myc* RNAi) GOF (UAS-*dmyc* )	Increases PSC cell number	(65)
Insulin/TOR	PSC	LOF (InR RNAi) GOF (UAS-PI3K ^CAAX^ )	Increases PSC cell number	(67, 68)
Bantam	PSC	LOF (UAS-sponge) GOF (UAS-*bantam* )	Increases PSC cell number	(69)
Bag of Marbles	PSC	LOF (*bam* RNAi)	Inhibits PSC cell proliferation	(70)
Thor/4EBP	PSC	LOF (*eIf4A* RNAi)	Increases PSC cell number	(70)
Retinoblastoma-family protein	PSC	LOF (*Rbf* RNAi) GOF (UAS-*Rbf* )	Inhibits PSC cell proliferation	(70)
ARF1-GTP	PSC and hemocytes	LOF (*arf1* RNAi)	Increases PSC cell number	(71)
Jumu	progenitors	LOF (*jumu* RNAi)	Inhibits PSC cell proliferation Promotes PSC cell clustering	(72)
Jumu	PSC	LOF (*jumu* RNAi) GOF (UAS-*jumu* )	Increases PSC cell number	(72)
Slit/Robo	PSC and cardiac cells	LOF (*robo* and *slit* RNAi)	Inhibits PSC cell proliferation Promotes PSC cell clustering	(73)
DE-cadherin	PSC	LOF (*DE-cad* RNAi)	Reduces PSC cell number Promotes PSC cell clustering	(73)
Cdc42	PSC	LOF (UAS-cdc42^DN^) GOF (*UAS*-cdc42^CA^	Increases PSC cell number Promotes PSC cell clustering	(73)
Coracle	PSC	LOF (*cora* RNAi)	Reduces PSC cell number	(74)
Neurexin IV	PSC	LOF (*nrxIV* RNAi)	Reduces PSC cell number	(74)
Lar	PSC	LOF (*Lar* RNAi) GOF (UAS-*Lar* )	Reduces PSC cell number	(75)
NUP98-HOXA9	PSC and hemocytes	GOF (UAS-NA9)	Promotes PSC cell proliferation	(76)
E2F	PSC	LOF (*E2F* RNAi)	Increases PSC cell number	(70)

The PSC requirement to control the balance between hemocyte differentiation and progenitor maintenance (homeostasis) in third instar larvae was first reported by Mandal et al. and Krzemien et al. In this context, Hedgehog (Hh) secreted by PSC cells is a key regulator of lymph gland homeostasis (63) and Col is required for PSC specification during embryonic development (64). It has been proposed that by controlling hematopoietic progenitor maintenance within the lymph gland, the PSC plays a role similar to a niche. However, several studies questioned the genuine interactions that take place between MZ and PSC cells, since ablation of PSC cells driven by the expression of the proapoptotic gene *reaper* (*rpr*) does not affect MZ progenitor maintenance but rather reduces crystal cell differentiation (78). Another study reported that reduction of PSC cell numbers or alteration of PSC signaling increases hemocyte differentiation without affecting the pool of “core progenitors”. Altogether, these two studies establish that core progenitors are maintained independently from the PSC [**Figure 2A** and (60, 78)]. More recent studies shed some light on these discrepancies. Baldeosingh et al. examined the effects on MZ progenitors of specific PSC cell ablation induced by *rpr* expression. For this, they analyzed the expression of Odd-skipped (Odd), a transcription factor expressed in all MZ progenitors. In the absence of PSC cells, a prohemocyte subpopulation with Odd-positive/Col-negative cells differentiated into mature hemocytes, whereas the Odd-positive/Col-positive cells remained undifferentiated (79). The study further reported that Hh from the PSC is required to maintain Odd-positive but not Col-positive prohemocytes, establishing that the MZ cell population is composed of Hh-independent (core progenitors) and Hh-dependent (distal progenitors) progenitors (**Figure 2A**). Altogether these data confirm that the PSC only regulates a subset of MZ progenitors and that this is achieved through Hh signaling.

Blanco et al. investigated the role of Ser in the PSC. *Ser* knockdown in PSC cells leads to increased plasmatocyte and crystal cell numbers, which is in agreement with previously published data (64). Furthermore, they report that Notch knockdown specifically in core progenitors leads to a reduction of their numbers. These data indicate that Ser in PSC cells restricts hemocyte differentiation [**Figure 2A** and (61)]. Ser requires cell-cell contact to activate the Notch pathway, raising the possibility that PSC filopodia could mediate Notch signaling, although the subset of progenitors controlled by Ser remains to be identified.

A recent study further established a role of the PSC in L1 larvae (80). At this stage the lymph gland is composed of PSC cells and hematopoietic progenitors and no differentiation occurs. The PSC counts 2-4 cells that express Col and Antp. Through the expression of different markers, it has been shown that two types of progenitors are present. One subset of progenitors, expressing Notch, is aligned along the cardiac tube, and this cell state is transient, since Notch positive cells are only found during the first 20 hours of larval development. Lineage tracing experiments established that this cell population gives rise to most lymph gland cells at later larval stages, leading the authors to propose that they correspond to genuine Hematopoietic Stem Cells (HSCs). The presence of these HSCs in L1 larvae is also niche-dependent. They rely on Dpp/BMP signaling issued from the PSC.

In summary, these studies reveal a temporal role for the PSC during larval development to regulate lymph gland hematopoiesis and further establish that different signals, long *versus* short distance, are produced by PSC cells throughout larval development to regulate different progenitor subsets. Thus, the lymph gland is a valuable model to investigate the spatial and temporal role of the niche. Additional analyses are required to identify other yet undetected PSC signals, define which progenitor sub-clusters respond to which PSC signals, and finally define how these various niche signals are integrated in progenitor subtypes to control the balance between progenitor maintenance and blood cell differentiation.

## The PSC Indirectly Controls Hematopoietic Progenitor Maintenance *via* Differentiated Hemocytes

In third instar larvae, the PSC indirectly controls hematopoietic progenitor maintenance *via* differentiated hemocytes. The PSC also secretes another ligand called PDGF and VEGF-related factor 1 (Pvf1), which binds and activates the Pvr tyrosine kinase receptor. Pvf1 is produced by PSC cells and transported by vesicles into CZ cells that express Pvr. Pvr activation in the CZ induces a Stat92E-dependent but JAK-independent signaling cascade, leading to the overexpression of adenosine deaminase-related growth factor A (Adgf-A). Stat92E activation is dependent on the ARF1/Asrij complex that encodes a ras small GTPase and an endocytic protein (71). Adgf-A downregulates adenosine levels in neighboring MZ cells, leading to a reduced activity of PKA (cAMP-dependent protein kinase 1). PKA controls the degradation of active Cubitus interruptus (Ci), the transcription factor mediating Hh signal transduction. This backward signal to the MZ is called the “equilibrium signal” (81, 82). Overall, signals from CZ and PSC cells regulate the balance of Ci activity within the MZ, thereby controlling progenitor maintenance (**Figure 2A**).

## The Cardiac Tube Functions as a Hematopoietic Niche

Within the MZ, “core progenitors” that express *col* and *tep4*, are in close contact with the cardiac tube and are maintained independently from PSC activity [**Figure 2A** and (60, 78, 79)]. This raises the possibility that cardiac cells contribute to the regulation of lymph gland homeostasis. Two recent studies investigated the role of the cardiovascular system under homeostatic conditions and established that cardiac cells act both i) indirectly *via* the PSC, and ii) directly on MZ progenitors to control lymph gland hematopoiesis. An initial study showed that the *Drosophila* cardiac tube is required to maintain the integrity and function of the PSC through Slit/Robo signaling. The Slit ligand secreted by cardiac cells activates Robo signaling in the PSC. Slit/Robo activation controls both the number of PSC cells and their cohesion, and in turn PSC function (**Table 1**). It controls PSC size by repressing BMP signaling, and maintains PSC cell clustering by regulating the activity of the Cdc42 small GTPase and the accumulation of DE-Cadherin. This study was the first to highlight an inter-organ communication between the cardiac tube and the lymph gland in order to control PSC morphology and consequently its function [**Figure 2B** and (73)]. In a second study, which investigated whether cardiac cells can directly act on MZ progenitors *via* secreted signals, the authors performed a candidate RNAi screen in cardiac cells to identify new potential signaling pathways involved in the crosstalk between the vascular and the hematopoietic systems. This study provided evidence that cardiac cells play a role similar to a niche through the activation of Fibroblast Growth Factor (FGF) signaling. The FGF ligand Branchless (Bnl) secreted by cardiac cells was detected in MZ progenitors as cytoplasmic punctate dots; it is internalized by MZ cells, most likely through FGF-receptor-mediated endocytosis. Bnl binding to its receptor Breathless (Btl) leads to Bnl/Btl-FGF pathway activation in MZ progenitors, where it controls calcium levels *via* the activation of phospholipase C (PLCγ). A previous study showed that reduction of cytosolic Ca^2+^ in lymph gland progenitors leads to the loss of progenitor markers and to increased blood cell differentiation (83). Altogether, these data indicate that through the activation of Fibroblast Growth Factor (FGF) signaling, the vascular system prevents hematopoietic progenitors from massive differentiation, ensuring the proper balance between blood cell populations within the lymph gland. For the first time, this study provides evidence that the vascular system, which directly controls blood cell progenitors independently from the PSC, acts as a niche [**Figure 2B** and (84)]. In conclusion, two distinct niches, the PSC and the cardiac tube, control lymph gland homeostasis.

## Emergency Hematopoiesis: Key Role for the PSC

**Figure 3:**
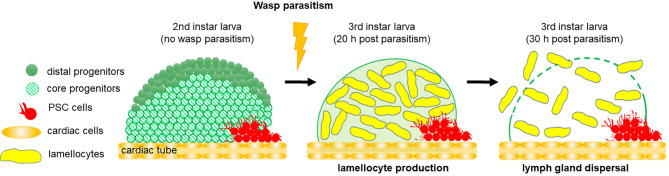
Lymph gland response to wasp parasitism. Schematic representation of 2^nd^ instar larval lymph gland, composed of PSC cells and progenitors. Twenty hours post parasitism, lamellocytes differentiate at the expense of progenitor maintenance. Thirty hours post parasitism, the lymph gland disrupts and cells are released into the hemolymph, where they encapsulate the wasp egg.

**Figure 4:**
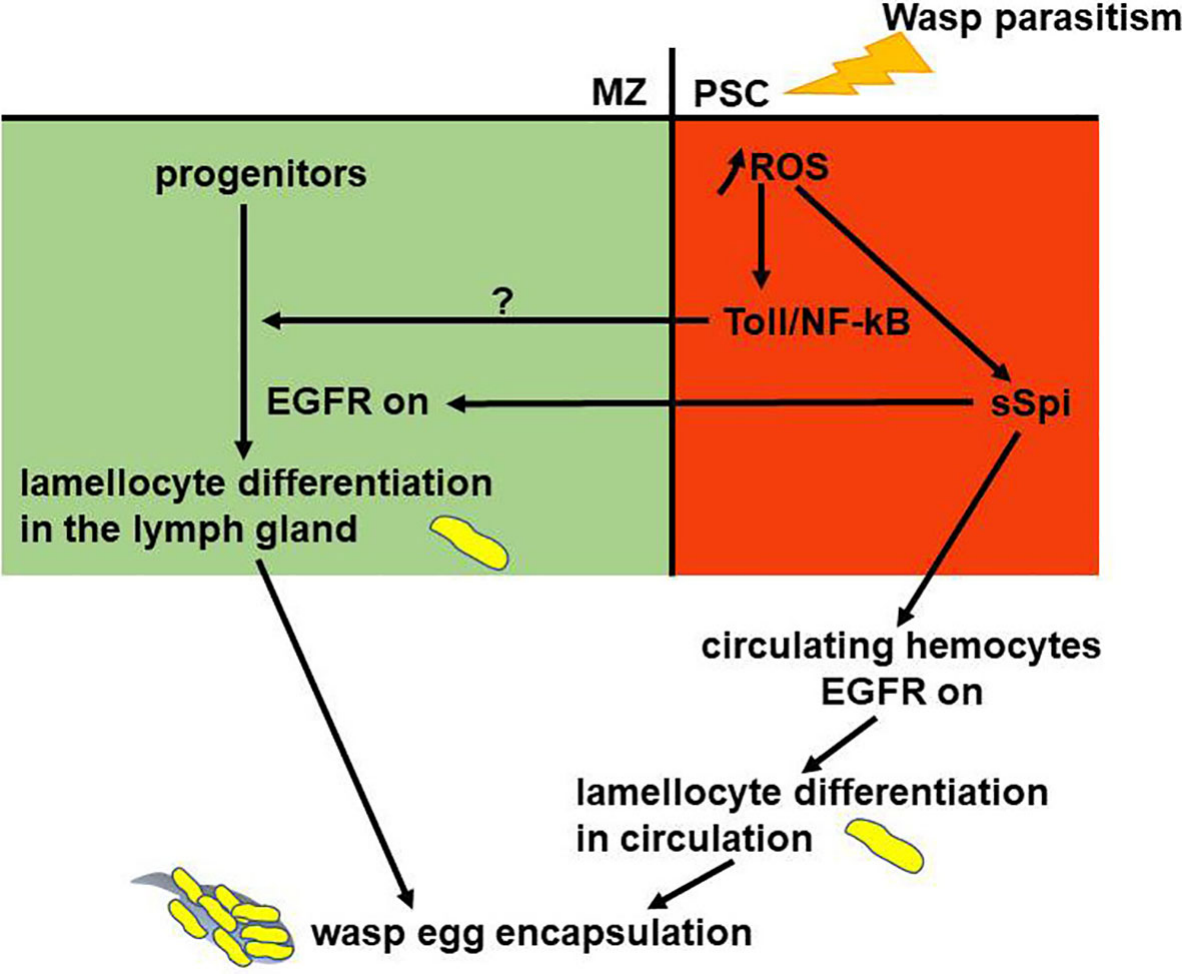
Gene regulatory network controlling larval emergency hematopoiesis. The PSC (red) plays an essential role in mounting the cellular immune response. In response to wasp parasitism, increased Reactive Oxygen Species (ROS) levels in the PSC cause lamellocyte differentiation from lymph gland progenitors (green) and circulating hemocytes. ROS in PSC cells activate Toll/NF-kB and Spitz secretion (sSpi). sSpi, the EGFR ligand, induces lamellocyte fate. Toll/NF-kB activation in the PSC regulates non cell-autonomously lamellocyte differentiation in the lymph gland. EGFR and Toll/NF-kB activation are required to regulate lymph gland stress hematopoiesis.

*Drosophila* blood cells are the effectors of the cellular arm of the innate immune response (24). Wasp parasitism is commonly used to induce an emergency hematopoiesis, which culminates in the massive differentiation of lamellocytes, a cryptic blood cell type (29, 85). Lamellocytes are specialized hemocytes, which mediate the encapsulation and killing of pathogens too large to be phagocytosed. Resistance to wasp parasitism depends on the ability of the *Drosophila* larva to reroute basal hematopoiesis and produce lamellocytes, in a timely manner, to neutralize wasp eggs before they hatch inside the fly larva. Following wasp egg-laying in a *Drosophila* second instar larva, the egg is identified as a foreign body and differentiation of lamellocytes from lymph gland MZ progenitors and circulating/sessile hemocytes is triggered (29, 32, 53–57). In response to wasp parasitism, lymph gland hematopoiesis is drastically modified and shifts to emergency hematopoiesis, leading to increased progenitor proliferation 4-6 hours post-parasitism (59, 86). 20 hours post parasitism, lamellocytes massively differentiate at the expense of MZ progenitors, ultimately leading to the premature dispersal of lymph gland anterior lobes [**Figure 3** and (86)]. The PSC is absolutely required for this emergency response, since lamellocytes fail to differentiate when PSC cells are ablated by targeted expression of *rpr* (60, 64, 78). It has been shown that in *Drosophila* larvae, parasitization increases Reactive Oxygen Species (ROS) levels in PSC cells, leading to the secretion of Spitz (sSpi), one ligand of the Epidermal Growth Factor Receptor (EGFR) signaling pathway (87). Spi issued from the PSC activates the EGFR pathway, both in circulating embryo-derived hemocytes and in MZ progenitors, which triggers their differentiation into lamellocytes. [**Figure 4** and (86, 87)]. Furthermore, it has been established that the Toll/NF-κB pathway is activated in PSC cells in response to wasp parasitism (86, 88). Activation of the pathway is triggered by high ROS levels in PSC cells, which leads to expression of Spätzle (Spz), the Toll/NF-κB pathway ligand, and subsequent activation of the pathway in the PSC. This pathway controls, in a non-cell autonomous manner, lymph gland lamellocyte differentiation in the MZ, which leads to premature disruption of lymph gland anterior lobes, and *in fine* successful wasp egg encapsulation by lamellocytes. It seems that in response to wasp infection, the EGFR and Toll/NF-κB pathways act in parallel to trigger lamellocyte differentiation from MZ cells [**Figure 4** and (86)]. How Toll/NF-κB activation in the PSC acts on MZ progenitors remains to be investigated.

JAK/STAT signaling is one of the evolutionarily conserved signaling pathways involved in immunity (89) and specifically in *Drosophila* for lamellocyte differentiation upon parasitism (90, 91). Under normal conditions, JAK/STAT is activated in lymph gland MZ hematopoietic progenitors; in response to wasp parasitism, the pathway is switched off in progenitors, thus triggering their differentiation into lamellocytes (90). Furthermore, wasp parasitism leads to JAK/STAT activation in larval somatic muscles, which in turn controls the number of circulating lamellocytes and the efficiency of wasp egg encapsulation (92).

In depth analysis of lymph gland hematopoiesis focuses on lymph gland anterior lobes. In response to wasp parasitism, hemocytes from posterior lobes do not differentiate into lamellocytes (23, 36, 58). The JAK/STAT pathway, which is activated in posterior lobes in response to parasitism, is required to prevent lamellocyte differentiation. Furthermore, while the PSC is essential in anterior lobes for the response to wasp parasitism, it plays no role in posterior lobes to prevent lamellocyte differentiation. Altogether, these data indicate a differential response to parasitism between anterior and posterior lobes. Finally, under wasp infection, cell coalescence is observed in posterior lobes, and this response is prevented when the PSC is ablated, suggesting a role for the PSC in this response (58). In conclusion, a complex regulation of JAK/STAT signaling is induced in response to wasp parasitism, and whether JAK/STAT activity in the different cell types could depend on the two niches, namely PSC or/and cardiac tube, certainly deserves further investigation.

Besides wasp parasitism, *Drosophila* can be infected by bacteria, fungi or viruses, which activate the humoral response (24). Interestingly, a recent study established that bacterial infection also alters lymph gland hematopoiesis, since it reported increased plasmatocyte and crystal cell differentiation at the expense of MZ progenitors upon infection. However, in contrast to wasp parasitism, no lamellocytes differentiated. The study further showed that septate junctions form a permeability barrier at the PSC that is disrupted following bacterial infection that trigger prohemocyte differentiation probably by enabling PSC signals to extend into the MZ (74). The authors further established that activation of the Toll/NF-κB and Immune Deficiency (Imd) pathways in PSC cells leads to the loss of the PSC permeability barrier. However, whether bacterial infection disrupts the niche permeability barrier *via* the activation of NF-κB pathways in the PSC is not known yet. Since the Toll/NF-κB pathway is activated in PSC cells and is required for lamellocyte differentiation, it is possible that the permeability barrier modification in PSC cells in response to wasp parasitism contributes to niche hematopoietic progenitor signaling. These data open novel insights into the cellular communication between the PSC and MZ progenitors.

In mammals, systemic bacterial infection activates the Toll/NF-κB pathway in mouse bone marrow endothelial cells, provoking an “emergency granulopoiesis” (93, 94). This, again, underlines the evolutionary conservation of molecular mechanisms controlling stress-induced hematopoiesis between *Drosophila* and mammals. As a conclusion, our comprehension of the mechanisms regulating emergency hematopoiesis in *Drosophila* should improve our fundamental understanding of how inflammatory signaling regulates hematopoiesis in health and disease conditions.

## Conclusions and Perspectives

*Drosophila* is a powerful *in vivo* model system to study the dialogue between a hematopoietic niche and progenitor cells, since several signaling pathways and transcription factors involved in the *Drosophila* microenvironment play comparable roles in mammals. Under homeostatic conditions, the transcription factor Col/EBF, expressed in PSC cells, is required for PSC specification (57, 64) and controls PSC cell numbers and function through BMP/Dpp pathway activation (65). In mouse osteoblasts, EBF2 is an essential component of the endosteal niche, where it controls osteoblast numbers and regulates HSPC maintenance (95, 96). The Notch pathway is also involved both organism. The Notch ligand Serrate is expressed in the *Drosophila* PSC, where it prevents progenitor differentiation (61, 62). Similarly, in mammalian osteoblasts, Notch1 and 3 and the ligands Jagged1 and Delta1 are all expressed and regulate hematopoiesis, although the precise regulatory mechanisms remain unclear (8, 97, 98). Furthermore, in *Drosophila*, Slit secreted by cardiac cells activates Robo receptors expressed by PSC cells. Silt/Robo activation controls PSC cell numbers and their function (73), while, in mouse bone marrow Slit2/Robo4 controls HSPC localization in the perivascular niche (99–101). In *Drosophila*, the FGF pathway is a key player in the communication between cardiac tube and hematopoietic progenitors. In mammals, this pathway remodels bone and the bone marrow microenvironment to support bone integrity, HSPC maintenance and expansion, and plays a crucial role for proper hematopoiesis during stress recovery (102). Finally, the high similarity between *Drosophila* and mammalian bone marrow hematopoiesis is further emphasized by our recent identification of the cardiac tube as a second niche for lymph gland hematopoiesis, reminiscent of the two niches, endosteal and perivascular, controlling HSPC self-renewal and differentiation in mammals.

Recent data based on single cell analysis revealed an unsuspected heterogeneity among lymph gland hematopoietic progenitors (41). Single-cell RNA sequencing performed on circulating *Drosophila* larval hemocytes highlighted a similarly unexpected heterogeneity among these cells, which were so far believed to consist of merely two cell types, crystal cells and plasmatocytes (40, 42, 103). These results raised many questions about the heterogeneity of the *Drosophila* blood cell pool and their regulation by different niche cell types. Likewise, in mammalian bone marrow, single cell approaches revealed a considerable heterogeneity among both niche and hematopoietic stem progenitor cells (10). Further analyses are now necessary to decipher which niche cells control which progenitor subset, to identify the signals involved in this crosstalk, and finally to determine how information provided by the diverse niche cells is integrated to control hematopoiesis under homeostatic conditions and after infection.

Mechanisms regulating emergency hematopoiesis are poorly understood. Oxidative stress regulates hematopoiesis *via* ROS both in Mammals and in *Drosophila* (87, 104, 105). In mammalian bone marrow, bacterial infection induces an “emergency granulopoiesis” that leads to *de novo* production of neutrophils. In this context, the TLR (Toll-like Receptor)/NF-κB pathway is activated *via* TLR4 in mouse bone marrow endothelial cells, a component of the vascular niche (93, 94). In *Drosophila*, the cellular immune response to parasitism is a typical emergency hematopoiesis. ROS levels increase in the PSC, thus activating both Toll/NF-κB and EGFR signaling pathways, which act in parallel to mount a stress hematopoiesis (86). Whether the EGFR pathway plays a role in mammalian hematopoiesis has not yet been established (106). Altogether, those studies are in favor of evolutionary parallels between *Drosophila* and mouse in the control of stress-induced hematopoiesis. The recent identification of the cardiac tube as a niche controlling lymph gland homeostasis under homeostatic conditions obviously raises the question about its potential role during emergency hematopoiesis.

Malignant hematopoiesis and inflammation in mammals is often associated with an abnormal microenvironment (2, 3, 12, 107, 108). Thus, deciphering the mechanisms at play in the HSPC/niche dialogue is of most importance and *Drosophila* stands as an invaluable model to do so. 

## Author Contributions

IM-P, YT, NV, and MC wrote the manuscript. IM-P and YT made the figures and NV the table. IM-P, YT, NV, and MC designed the review. All authors contributed to the article and approved the submitted version. 

## Conflict of Interest

The authors declare that the research was conducted in the absence of any commercial or financial relationships that could be construed as a potential conflict of interest.

## Publisher’s Note

All claims expressed in this article are solely those of the authors and do not necessarily represent those of their affiliated organizations, or those of the publisher, the editors and the reviewers. Any product that may be evaluated in this article, or claim that may be made by its manufacturer, is not guaranteed or endorsed by the publisher.
